# RDN for the treatment of influenza in children: a randomized, double-blinded, parallel-controlled clinical trial

**DOI:** 10.1186/s12906-023-04037-1

**Published:** 2023-07-20

**Authors:** Shuo Yang, Yan-ming Xie, Lian-xin Wang

**Affiliations:** 1 Institute of Basic Research in Clinical Medicine, China Academy of Chinese Medical Sciences, Beijing, China; 2 China Academy of Chinese Medical Sciences, NO. 16, Nanxiao Street, Inner Dongzhimen, Dongcheng District, Beijing, China

**Keywords:** Influenza in children, Reduning injection, Efficacy and safety, Time of temperature recovery

## Abstract

**Background:**

The morbidity of influenza in children increased rapidly in decade. Reduning injection (RDN), a small but fine Chinese herbal formula, has antipyretic, antiviral, anti-inflammatory effects. We intend to evaluate the efficacy and safety of RDN for the influenza in children versus Oseltamivir, explore the possible antiviral mechanism of RDN and provide evidence-based medical evidence for rational clinical drug usage.

**Method:**

We design a randomized, double-blind, double-dummy, parallel control of positive drug, multi-centre clinical study. According to the formula of mean superiority test, a total of 240 patients with influenza in children will be randomized 1:1 into the experimental group and control group. The experimental group will take RDN and Oseltamivir phosphate granule simulants and the control group will take Oseltamivir phosphate granule and RDN simulants. Each group will be treated for 5 days. The primary outcome measure is temperature recovery time, and the secondary outcome measures include time when the fever begins to subside, time and degree of disease to alleviate, disappearance rate of individual symptoms and so on. We will measure before enrollment and each 24 h after treatment for comparison.

**Discussion:**

The study is launched to evaluate the efficacy and safety of RDN for the treatment of influenza in children and to provide an alternative option for influenza in children.

**Trial registration:**

This study is registered in ClinicalTrials.gov as NCT04183725, registered on 3 December, 2019.

**Supplementary Information:**

The online version contains supplementary material available at 10.1186/s12906-023-04037-1.

## Introduction

Influenza, a negative-strand RNA virus of the Orthomyxoviridae family, is classified into influenza A, B and C viruses (IAV, IBV and ICV). Specially, with its “reassortment” of segmented genomes [1], IAV is easily to cause a pandemic in human. Influenza virus invades respiratory tract usually in winter leading to acute infectious disease accompanied by characteristic symptoms of fever suddenly, cough, chills or sweats, myalgia and malaise [2, 3]. Infectious disease was the most important cause of death in children under 5 years [4], and young children were the major targets of influenza comparing with older counterparts [5]. Children under 5 years had the highest rates of influenza-associated hospitalizations and Influenza-like illness (ILI) outpatient visits [6]. Besides, the age composition ratio from 2010 to 2019 of discharge patients in China indicated that influenza population under 14 years ranged from 33.3% grow into 78.5% [7]. Consequently, the direct and indirect burden on society and family has increased a lot compared to before [8].Therefore, one of the most urgent issues is how to response to influenza in children effectively to reduce the burden.

Fever, occurring in 95% of influenza children, is the most salient sign, 59% of children 3 years has fever 39.0℃ [9]. Fever outdoes all others and is the only reliable predictor of influenza virus infection [10]. We selected time of temperature recovery as the primary measure to evaluate treatment effect. Time when fever begins to subside is also included as a secondary outcome to observe when RDN starts to work. Apart from fever suddenly, clinical features of ILI such as cough, chills, running nose, sore throat, headache, myalgia and so on are common clinical manifestations [9, 11, 12]. So clinical symptom record table and score of each symptom are developed to record time and degree of disease alleviation to understand the change of disease. The disappearance rate of individual symptoms will be recorded after medication to evaluate RDN’s effect on each symptom. Although clinical manifestations are typical for diagnosis, laboratory testing is necessary too [13]. So we select rapid antigen test [14] to estimate negative conversion rate of influenza viral and detect its type [2]. Special circumstance such as symptomatic treatment needs to be considered, thus we will record number and frequency of antipyretic and analgesic drugs if using. Children < 5 years are likely to develop severe complications (age < 2 years easier) [15], hence we will monitor incidence of complications of influenza to ensure children’s safety.

Reduning (RDN), consisting of Qing Hao (*sweet wormwood herb*), Zhi Zi (*fructus gardeniae*) and Jin Yin Hua (*honeysuckle*), is a traditional Chinese medicine injection under patent protection (Chinese patent number CN201711264903.0). RDN treats influenza virus through alleviating pulmonary inflammation and reducing inflammatory factors release [16]. Meanwhile RDN has been recommended to treat H7N9 influenza infection [17]. A case of 108 patients of influenza in adult, RDN has a comparable effect with Oseltamivir but faster work and short course [18]. We intend to evaluate efficacy and safety of RDN to provide a possible therapeutic strategy for influenza in children. Oseltamivir (Kewei) is the recommended drug for influenza in children approved by *Influenza Diagnosis and Treatment Program (2018 Edition)* [15] and *Recommendations for Prevention and Control of Influenza in Children (2016–2017)* [19], so we choose it as positive control to reflect the superior curative efficacy and safety of RDN objectively.

## Method

### Study design

This is a randomized, double-blinded, double-dummy, parallel control of positive drug, multi-centre clinical study. The trial will be conducted in ten centers at China and collaborate with hospitals including Children’s Hospital Of Soochow University, Anhui Provincial Children’s Hospital, Qilu Children’s Hospital of Shandong University, Tianjin 4th Centre Hospital, Renmin Hospital of Wuhan University, Hebei Maternity&Child Healthcare Hospital, the Second Affiliated Hospital of Jiaxing University, the First Hospital of Hunan University of Chinese Medicine, Affiliated Hospital of Shanxi University of Traditional Chinese Medicine and one undetermined. The trial obtained ethical approval from Ethics Committee of The First Affiliated Hospital of Henan University of Chinese medicine (approval number: 2019HL-137-01), and will be conducted according to *the World Medical Association Declaration of Helsinki* and *Good Clinical Practice of Pharmaceutical Products* [20, 21].

**Fig. 1:**
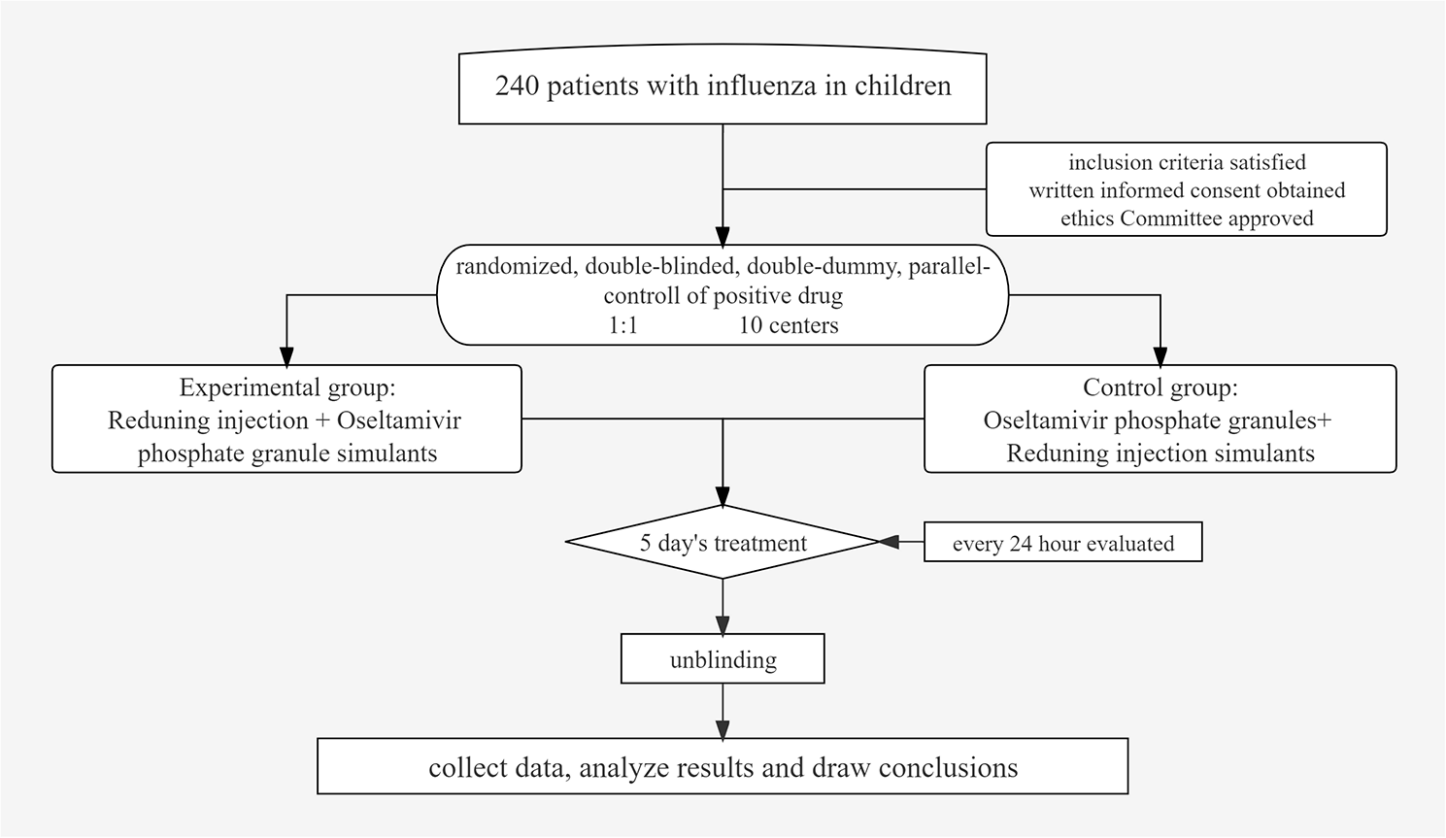
Flow diagram

Participants with influenza in Children will undergo a standardized baseline evaluation before treatment. All included participants are randomly divided into two groups: experimental group and control group.The experimental group receives RDN + Oseltamivir phosphate granule simulants and the control group receives Oseltamivir phosphate granules + RDN simulants. After five days of treatment with evaluation every 24 h, the efficacy and safety of two groups will be evaluated. The study design is shown in Fig. 1.

### Sample size estimation and randomization

Taking time of temperature recovery as clinical main efficacy indicator, we calculate two groups’ sample size using mean superiority test formula. According to data of efficacy evaluation about adult influenza treating with RDN and Oseltamivir in the early stage, the per protocol set (PPS) of time of temperature recovery comes: Oseltamivir is 46.50 ± 1.74(h) and RDN is 30.47 ± 1.69(h); so that δ for 15.35 h, 100 participants will be required for each group, and 200 is the total. If the 20% dropout rate is considered, 240 cases were planned to be observed. We adopt the method of center stratified block randomization in trial: there is 10 centers, 4 segments, each center has 24 cases and each group has 12 cases.

Randomization will be used for patient allocation [22]. The randomization will be performed by an independent statistician. The random numbers are divided into two groups sequentially: experimental group and control group. SAS 9.1.3 statistical software will be used for seed number, and randomized grouping tables will be generated for the 240 patients receiving treatment. During the trial, the investigator will be able to obtain the randomized number and drug number of each patient from the designated central randomized platform. All random numbers grouped are sent to each trial center and corresponding treatment kits are provided.

### Blinding

This is a double-blind (patients and clinicians are blinded) and double-dummy study. A two-stage blind design is adopted. The first stage was the group corresponding to number of each case (such as group A, group B) and the second stage was the intervention corresponding to each group (experimental group or control group).

Treatment assignments will not be revealed until the whole process is completed. Emergency letter will be opened by researchers when emergency (such as severe adverse events) or patient needs rescue and must know the treatment had received. Once the blind is broken, the patient with this number will be withdrawn from the trial handling as a dropout case and the researchers should record the reasons in the case report form (CRF). All emergency letters will be returned together with the CRF after the trial for blind review. When the blind codes are leaked or the opening rate of emergency letter exceeds 20%, the double-blind test will declare failure.

To achieve blinding, the outer packaging of experimental and control drugs should be identical. Size, color, shape, taste, smell and packaging of the simulant are identical to that of the corresponding medicine by adding artificial pigment [23].

### Eligibility criteria

The diagnostic criteria will be formulated from epidemiological history, clinical manifestations and laboratory diagnosis according to *the Guidelines for the diagnosis and treatment of Influenza (Beijing 2018)* [15].

### Inclusion criteria

1. Patients get influenza within 48 h with positive results of rapid defection for virus antigen.
2. Apart from general influenza symptoms, patients’ temperature before study is over 38℃;
3. Patients’ age is between 2 and 14 years and informed consents (from guardian or patient themselves) is got.

### Exclusion criteria

1. Patients is not common influenza (severe or critical) or concomitant other respiratory or pulmonary infections;
2. Patients is not virus infection (mycoplasma infection or bacterial infection);
3. Patients’ laboratory examination results such as serum creatinine (SCR), alanine aminotransferase (ALT) or aspartate aminotransferase (AST) are exceeded the normal range;
4. Patients has severe malnutrition, immunodeficiency (or taking glucocorticoid) or severe vital organ diseases;
5. Patients with severe infections must be treated with other antiviral drugs;
6. Patients are allergic to the RDN or Oseltamivir phosphate granules;
7. Patients has taken Oseltamivir or other heat-clearing & detoxicating traditional Chinese medicine before study;
8. Patients may cause loss of follow-up such as unstable living environment.

Patients with any of the above should be excluded.

### Exit criteria

Patients will leave the trial when one of the following criteria is met:

1. The symptoms of patients get worse after 3 days of medication;
2. Some comorbidities, complications or special physiological changes occur during the test;
3. Patients with poor compliance don’t reach 80% of the prescribed amount should be used in the trial;
4. Cases breaking the blind or opening the blind urgently in the trial;
5. Drugs out of prescribed protocol are used in the trial;
6. Patients who want to quit the trial or refuse to accept prescription and examination have the right to withdraw from the trial according to the provisions of the informed consent. And the reason should be understood and documented possibly.

Notice: Whatever reason the cases are dropped out of the trial, the CRF should be kept and transformed into final result using the last result examined, and the efficacy and adverse reactions (ADRs) should be carried out using its full data set.

#### Suspension criteria

1. Serious problems about safety occur in the trial;
2. The curative efficacy of drugs is too poor or even ineffective and has no clinical value in the trial;
3. Severe mistakes of clinical trial protocol are founded that is difficult to evaluate the efficacy of the drug; or obvious deviation occur in the progress of implementation and it is difficult to evaluate efficacy of the drug if it continues;
4. The sponsor requests the suspension (such as financial reasons, management reasons, etc.);
5. The administrative department cancels the test.

### Elimination criteria

1. Patients do not meet the inclusion criteria are founded after enrollment;
2. Drugs out of prescribed plan are used in the trial;
3. Cases no medication after enrollment;
4. Cases no evaluation or record about medication.

### Informed consent

The participants are between 2 ~ 14 years old. After a full explanation by the clinicians, written informed consent from participants (or their parent or legal guardian in the case of children under 16) will be obtained before intervention [24, 25].The doctor should describe the purpose, process, and likely risks of this trial in writing to the patient or their guardian before patients enrolled in the trial. Every patient has the right to withdraw from the trial at any time. Written informed consent will be kept in the study file.

### Interventions

All researchers are clinical doctors and they will receive standardized training before starting trial. The experimental drug is RDN and the control drug is Oseltamivir. RDN simulant and Oseltamivir simulant have no active ingredients, the former is intravenous drip with normal saline (NS) or grape sugar (GS) avoiding light and the latter is identical with Oseltamivir’s color, flavor and appearance. The detailed method of administration of two groups is shown in Table 1. And the curative course is five day and each 24 h has an evaluation.

**Table 1:** The detailed method of administration of two groups

	Age/Weight	Administration
**RDN and its simulant**	2 ≤ Age ≤ 5 years	0.5ml/kg/day, maximum is no more than 10ml/day 5%GS/0.9%NS diluted 50 ~ 100ml iv drip 30 ~ 40gtt/min q.d.*5
	6 ≤ Age ≤ 10 years	10ml/day 5%GS/0.9%NS diluted 100 ~ 200ml,iv drip, 30 ~ 60gtt/min q.d.*5
	11 ≤ Age ≤ 13 years	15ml/day 5%GS/0.9%NS diluted 200 ~ 250ml,iv drip, 30 ~ 60gtt/min q.d.*5
	Age = 14 years	20ml/day 5%GS/0.9%NS diluted 250ml,iv drip, 30 ~ 60gtt/min q.d.*5
**Oseltamivir and** **its simulant**	Weight ≤ 15 kg	30 mg, p.o. b.i.d.*5
	15<Weight ≤ 23 kg	45 mg, p.o. b.i.d.*5
	23<Weight ≤ 40 kg	60 mg, p.o. b.i.d.*5
	Weight>40 kg	75 mg, p.o. b.i.d.*5

### Discontinuation

Clinical symptoms and signs have no improvement or aggravation compared with those before enrollment after taking the medicine for 3 days or more. That needs to switch to another treatment and stop the medication. After completing the post-treatment evaluation and related laboratory tests, this case is declared over and this patient is recorded in PPS as invalid and qualified case statistics. When the body temperature of patient returns to normal and the symptoms and signs disappear within 5 days, the drug can be stopped. Follow-up visit is conducted after 24 h. If there is no recurrence, it will be counted as a recovered case.

### Concomitant

Drugs out of curative plan such as heat-clearing and detoxicating traditional Chinese medicine and oral or intravenous hormone that maybe affect the effect of Oseltamivir shall not be used. When taking antipyretic and analgesic drugs necessarily, the usage and dosage of antipyretic and analgesic drugs are recorded as one of the secondary efficacy evaluation indicators. Concomitant medication is required due to adverse events during the trial, which should be recorded in the CRF in detail.

### Follow-up

All included patients will be re-evaluated after five days of treatment to assess the efficacy and safety. Patients whose symptoms worsened will receive a supply of relevant medicine and a written withdrawal schedule.

### Outcomes

By searching for relevant literature [26–28] and interviewing relevant clinical experts, we observed the efficacy from five dimension: temperature, symptoms, use of antipyretic analgesics, etiological test and inflammatory factor test.

### Primary outcome measure

Time of temperature recovery.

1. Definition: time when axillary temperature drops below 37.3℃ without repetition after the first use of the drug.
2. Regulation of axillary temperature recording (Mercury thermometer and body temperature measuring instrument): after the first usage in 24 h, the temperature will be measured in the first 2 h, 4 h, 6 and 8 h; and after 24 h, the temperature will be measured at least 2 times a day. When axillary temperature drops below 37.3℃ without repetition in 24 h, it is the treatment end point.

### Secondary Outcome measure

1. In clinical manifestation, we will record such as time point of fever subside, time point of disease alleviate, degree of disease remission and disappearance rate of individual symptoms;
2. In laboratory examination, we will record the rate of negative conversion of Influenza viral;
3. In treatment, we will record the usage and frequency of antipyretic and analgesic drugs;
4. In outcome, we will record the incidence of complications of influenza.

### Criteria of comprehensive efficacy

Criteria of cure: the curative effect of clinical symptoms reaches the level of cure after finishing medication and the rapid detection for virus antigen of influenza turns negative. All two criteria must be met.

Criteria of invalid treatment: the curative effect of clinical symptoms is invalid, aggravated or complicated after the end of the medication and the rapid detection for virus antigen of influenza is still positive. Only one of the above is satisfied, it is considered invalid.

### Safety assessments

All participants will undergo the following laboratory examination. Safety assessments include vital signs, blood routine, urine routine, liver function, kidney function and twelve-lead electrocardiogram (ECG). We will judge anaphylaxis according to *criteria for diagnosis of anaphylaxis* by National Institute of Allergy and Infectious Diseases/Food Allergy and Anaphylaxis Network [29]. Then all adverse event (AE) will be judged in order to isolate ADR according to *Administrative Measures for the Reporting and Monitoring of Adverse Drug Reaction* [30] issued by the Drug Evaluation Center of the State Food and Drug Administration. And the Medical Dictionary for Regulatory Activities (MedDRA) will be used for standardization of ADR.

Assessment of study endpoints is shown in Table 2. It adheres to the Standard Protocol Items: Recommendations for Interventional Trials Statements (SPIRIT) [31].

**Table 2:** Measurement items and assessment of study endpoints

Study period	Lead-in period	Treatment period				
**Follow-up times**	1	2	3	4	5	6
**Time point**	0 Day	24 h	48 h	72 h	96 h	120 h
**Case screening**						
History	√					
Indicator check	√	√				√
Basic information	√					
Determine the inclusion	√					
Informed content	√					
**Indicator check**						
Temperature	√	√	√	√	√	√
BP/HR/P/symptoms/signs	√	√	√	√	√	√
Blood routine/Urine routine	√					√
Liver function/Kidney function	√					√
Twelve-lead ECG	√					√
Pathogen test	√					√
Inflammatory factor test	√					√
**Others**						
randomization	√					
Drug distribution	√					
Drug recycle/ medication compliance						√
Combined medication		√	√	√	√	√
AEs		√	√	√	√	√
Recycle drug						√
**Conclusion of Completion of experiment**						√

### Data management

CRF will be written by researcher and that will be delivered to the specific data administrators to establish a database after passing the examination of the clinical supervisor. The data on CRF is inputted into the database by two independent data administrator respectively which is reviewed manually, checked by computer, examined blindly, and then is locked for the statistical analysis.

### Data statistic and analysis

#### Data selection

Four datasets will be conducted intention to treat (ITT), full analysis sets (FAS), per protocol set (PPS), and safety analysis set (SS). ITT is conducted for efficacy and AEs. FAS refers to the ideal set of participants who are as close as possible to the principle of intention to treat and is obtained after removing the participants from all the randomization with the least and reasonable method. PPS, a subset of FAS, is a data set generated from disease case set fully compliant with the trial protocol. SS refers to the randomized cases that have taken a tested drug at least once with safety evaluation data after treatment.

### Statistical method

Data analysis will be performed by professional statisticians using SAS 9.1.3. For measurement data, Group t test or Wilcoxon rank sum test will be used for comparison between groups; for counting data, χ^2^ test and Fisher exact test will be used, Rank sum test and Cochran Mantel Haenszel (CMH)test will used for grade data; for efficacy indicators, PPS analysis and FAS analysis will be used at the same time. The statistical analyses will use the two-sided hypothesis test. P ≤ 0.05 will be set as the significant level.

### Analysis contents

1. List the frequency of cases enrolled, violated and dropped out;
2. List the details of cases of violation, dropout, low compliance, time-window violation, out of protocol drug usage.
3. List the datasets for analysis.
4. Comparability analysis: compare demographic data and other baseline indicators to measure the balance of each group.
5. Effectiveness analysis: FAS and PPS analysis. The study is a multi-center clinical trial, the influence of central effect on efficacy indicators should be considered in the analysis. Measurement data uses covariance analysis which considering the central factor, and count data uses CMH test corrected by the central stratification.
6. Safety analysis: compare the incidence of AE in each group and describe the AE in the trial. Compare the changes of normal/abnormal laboratory indicators before and after the test, and list the details of cases with abnormal/abnormal aggravation before and after the test.
7. Concomitant medication analysis: compare the details of each group and list them down.

### Quality control

#### Measures of quality control

Sponsor and researchers will adopt standard operating procedures (SOP) to ensure implementation of quality control and quality assurance systems for clinical trials. We will verify all observations and findings to make sure each conclusion in clinical trial deriving from original data. Meanwhile we will conduct quality control in every stage to acquire reliable and correct data.

#### Training of investigators

Supervisor with each sub-center will conduct clinical trial training for researchers before starting trials. They will know experimental drug’s nature, function, efficacy and safety (including related information before marketing), and any new information about the drug that finding during the trial.

#### Enhancement of compliance of patients

1. Researchers will conduct informed consent carefully to ensure subjects understand experimental requirements fully and comply to trial. They should know that the sponsor will provide free trial medication, laboratory examination fees and so on.
2. Using dose counting method monitors subjects compliance. Compliance = actual dosage/prescribed dosage × 100%. Good drug compliance: 80 − 120%; poor drug compliance: less than 80% or more than 120%.
3. Researcher should require subjects to bring all drugs when follow-up and record on CRF. Patients with poor efficacy and compliance will strengthen follow-up.

#### Monitoring of clinical trials

The sponsor appoints supervisor. Supervisor will visit trial hospitals on-site regularly to guarantee clinical protocol’s implementation strictly, and check original data to ensure it is identical with CRF.

#### Audit of clinical trials

The drug supervision and administration department and sponsor will entrust inspectors to conduct inspection systematically to ensure the execution of the trial consistent with protocol, and the data reported by the sub-center is identical with CRF or other original records. The audit will be carried out by personnel who is not directly involved in the clinical trial.

## Discussion

Influenza is getting more and more serious these years and early antiviral treatment can maximize clinical benefits [32]. Oseltamivir is the recommended drug for influenza in children [19]. We don’t know whether there are other drugs can play a better role in treatment for influenza in children. So we designed a study to evaluate the efficacy and safety of RDN for influenza in children comparing with Oseltamivir. 240 patients were divided into experimental group and control group in a randomized, double-blinded, double-dummy, parallel control of positive drug, multi-center clinical trial. Furthermore, we would explore the antiviral mechanism of RDN exactly and provide evidence-based medical evidence for clinical rational usage.

There are two limitations in the study. One is that children cannot express their discomfort symptoms well; another is the results of clinical symptom record table might have subjective factors.

## Electronic supplementary material

Below is the link to the electronic supplementary material.

Supplementary Material 1

## Data Availability

The data will be available when collected.

## Declarations

### Trial status

The study is currently at the systematic literature review stage.

## Abbreviations

RDN: Reduning injection
ILI: Influenza-like illness
ADR: adverse reaction
AE: adverse event
NS: normal saline
GS: grape sugar
ECG: electrocardiogram
MedDRA: the Medical Dictionary for Regulatory Activities
BP: blood pressure
R: heart rate
P: pulse
CRF: case report form
PPS: per protocol set
ITT: intention to treat
FAS: full analysis sets
SS: safety analysis set
CMH: Cochran Mantel Haenszel
SOP: standard operating procedure
