# Supplementary material for: RDN for the treatment of influenza in children: a randomized, double-blinded, parallel-controlled clinical trial
Source: BMC Complement Med Ther. 2023 Jul 20;23:255. doi: 10.1186/s12906-023-04037-1 (PMC10357598; doi:10.1186/s12906-023-04037-1)
Supplement: Supplementary file 1 — Supplementary Material 1 [file 12906_2023_4037_MOESM1_ESM.pdf]

SOP for Collection and Transportation of Clinical Samples for Influenza Detection

Collection of influenza clinical samples

The success of virus isolation depends to a large extent on the quality of clinical specimens and the links of preservation and transportation. Most of the specimens were taken from the upper respiratory tract and nasopharynx, followed by trachea and bronchial secretions and autopsy tissues. Samples should be put into appropriate sampling solution for low-temperature storage immediately after collection.

The common sampling solutions include the following five kinds: common broth, Hank's, Eagle's with pH 7.4~7.6, hydrolyzed milk protein solution or physiological saline without antibiotics (throat rinse). In order to prevent the growth of bacteria and fungi in the sampling solution, it is necessary to add antibiotics, gentamicin, with the final concentration of 0.1mg/ml, and antifungal drugs, with the final concentration of 2ug/ml. After adding the antibiotic, readjust the pH value to 7.4. After preparation, separately pack 4ml of each sampling tube and freeze it at - 20 ℃. There are several sampling methods:

Nasal swab: insert the swab with polypropylene fiber head parallel to the upper jaw into the nostril for several seconds. After the swab head absorbs the secretion, slowly turn and exit. Wipe the other nostril with another swab. Immerse the swab head into the sampling solution and discard the tail.

Pharyngeal swab: use a swab with polypropylene fiber head to wipe the bilateral pharyngeal tonsils and posterior pharyngeal wall with moderate force to avoid touching the tongue. Also immerse the swab head in the sampling solution and discard the tail. Note: Nasal and pharyngeal swabs can also be collected in the same sampling tube to improve the separation rate and reduce the workload.

Packaging and transportation of clinical specimens

1.It is recommended to use plastic tube with screw mouth for clinical sample collection tube and tighten it. There should be no leakage after the cover is closed. Write the name and number of the case directly on the plastic tube with an oily marker.

At the same time, fill in the relevant information of the specimen in the "inspection form", which should be separately placed in a waterproof bag. Put the sealed specimen into a plastic bag with biosafety signs of suitable size to seal.

2.Put the sealed bag containing the sample into the special transport box (or vaccine refrigeration bag), put it into the ice row, and then fill it with soft materials, lined with materials with water absorption and buffering capacity.

3.Fresh clinical samples should be transported to the influenza detection laboratory within 24 hours under 4 ℃. If it is not delivered to the laboratory within 24 hours, it shall be stored at - 70 ℃ or below. Frozen clinical samples should be sent to the laboratory at low temperature under frozen conditions. Frozen samples can be stored at 4 ℃ for virus isolation within 24 hours after being sent to the laboratory. If the sample cannot be separated, it should be stored at - 70 ℃ or below.
